# Kindness the Best Punishment

**Published:** 1880-05

**Authors:** 


### KINDNESS THE BEST PUNISHMENT.

A Quaker of most exemplary character, having been disturbed one night by footsteps around his dwelling, rose from his bed, and cautiously opened a back door to reconnoitre. Close by was an outhouse, and under it a cellar, near a window of which was a man busily engaged in receiving the contents of his pork barrel from another within the cellar. The old man approached, and the man outside fled. He stepped up to the cellar window and received the pieces of pork from the thief within, who, after a little while, asked his supposed accomplice, in a whisper, "Shall we take it all?" The owner of the pork said softly, "Yes, take it all;" and the thief industriously handed up the balance through the window, and then came up himself. Imagine his consternation, when, instead of greeting his companion in crime, he was confronted by the Quaker. Both were astonished; for the thief proved to be a near neighbor, of whom none would have suspected such conduct. He pleaded for mercy, begged him not to expose him, spoke of the necessities of poverty, and promised faithfully never to steal again. "If thou hadst asked me for the meat," said the old man, "it would have been given thee. I pity thy poverty, and thy weakness, and esteem thy family. Thou art forgiven." The thief was greatly rejoiced, and was about to depart, when the old man said, "Take the pork, neighbor." "No, no," said the thief, "I don't want the pork." "Thy necessity was so great that it led thee to steal. One-half of the pork thou must take with thee." The thief insisted that he could never eat a morsel of it. The thoughts of the crime would make it choke him. He begged the privilege of letting it alone. But the old man was inflexible, and, furnishing the man with a bag, had half the pork put therein, and laying it upon his back, sent him home with it. He met his neighbor daily for many years afterwards, and their families visited together, but the matter was kept secret; and though in after years the circumstance was mentioned, the name of the delinquent was never made known. The punishment was severe and effectual. It was probably his first—it was certainly his last attempt to steal. Had the man been arraigned before a court of justice, and imprisoned for the petty theft how different might have been the result! His family disgraced, their peace destroyed, the man's character ruined, and his spirit broken. Revenge, not penitence, would have swayed his heart. The scorn of the world would have blackened his future, and in all probability he would have commenced a course of crime at which, when the first offence was committed, his soul would have shuddered. And what would the owner of the pork have gained? Absolutely nothing. Kindness was the best punishment, for it saved while it punished.

---

Grass in Rum.—An old fellow in Missouri, who was in the habit of "not belonging to the Temperance Society," was in the act of taking a nip one day before a young Virginian.

"What do you drink?" asked the latter.

"Brandy and water," was the reply.

"Why don't you drink mint juleps?"

"Mint juleps?" queried the old man, "why, what in the name of drink is that?"

"A most delicious drink," was the answer; "and I'll show you how to make it, as I see you have mint growing almost at your door."

The young fellow soon produced the juleps, and the old man was delighted with it.

About a month after, on his return home, the Virginian thought would stop at his old friend's and "indulge," but judge of his surprise when his inquiries at the door for his friend was answered by an aged female darkey, with:

"Oh, Massa's dead and gone dis two weeks!"

"Dead!" exclaimed the young man, "why, how strange! What did he die of?"

"Oh, I d'no," returned the woman, "only a fellow come along about a monf ago and larnt him to drink *grass in he rum*, and it killed him in two weeks."

---

Love that has nothing but beauty to keep it in good health, is short-lived, and apt to have ague fits.

The parent who would train up a child in the way he should go, must go in the way he would train up his child

